# Glucose enhances indolic glucosinolate biosynthesis without reducing primary sulfur assimilation

**DOI:** 10.1038/srep31854

**Published:** 2016-08-23

**Authors:** Huiying Miao, Congxi Cai, Jia Wei, Jirong Huang, Jiaqi Chang, Hongmei Qian, Xin Zhang, Yanting Zhao, Bo Sun, Bingliang Wang, Qiaomei Wang

**Affiliations:** 1 Key Laboratory of Horticultural Plant Growth, Development and Quality improvement, Ministry of Agriculture, Department of Horticulture, Zhejiang University, Hangzhou 310058, China; 2 Zhejiang Provincial Key Laboratory of Horticultural Plant Integrative Biology, Department of Horticulture, Zhejiang University, Hangzhou 310058, China; 3 National Key Laboratory of Plant Molecular Genetics, Institute of Plant Physiology and Ecology, Shanghai Institute for Biological Sciences, Chinese Academy of Sciences, Shanghai 200032, China

## Abstract

The effect of glucose as a signaling molecule on induction of aliphatic glucosinolate biosynthesis was reported in our former study. Here, we further investigated the regulatory mechanism of indolic glucosinolate biosynthesis by glucose in *Arabidopsis*. Glucose exerted a positive influence on indolic glucosinolate biosynthesis, which was demonstrated by induced accumulation of indolic glucosinolates and enhanced expression of related genes upon glucose treatment. Genetic analysis revealed that MYB34 and MYB51 were crucial in maintaining the basal indolic glucosinolate accumulation, with MYB34 being pivotal in response to glucose signaling. The increased accumulation of indolic glucosinolates and mRNA levels of *MYB34*, *MYB51*, and *MYB122* caused by glucose were inhibited in the *gin2-1* mutant, suggesting an important role of HXK1 in glucose-mediated induction of indolic glucosinolate biosynthesis. In contrast to what was known on the function of ABI5 in glucose-mediated aliphatic glucosinolate biosynthesis, ABI5 was not required for glucose-induced indolic glucosinolate accumulation. In addition, our results also indicated that glucose-induced glucosinolate accumulation was due to enhanced sulfur assimilation instead of directed sulfur partitioning into glucosinolate biosynthesis. Thus, our data provide new insights into molecular mechanisms underlying glucose-regulated glucosinolate biosynthesis.

Glucosinolates (GS) are a group of nitrogen- and sulfur-containing secondary metabolites found throughout the family Crucifereae. Glucosinolates and their degradation products have a wide range of biological functions, including their well-known roles in plant defense against generalist herbivores, feeding or oviposition preference of crucifer-specialist herbivores^1^,^2^,^3^ as well as inhibition of microbial growth^4^,^5^. In addition, they also serve as important flavor components and anticarcinogenic agents^6^,^7^.

Glucosinolates are derived from amino acids, and can be grouped into aliphatic, aromatic, and indolic glucosinolates depending on the characteristic of the amino acids they originate from. The main biosynthetic pathway of glucosinolates has been elucidated in *Arabidopsis*. For example, in indolic glucosinolate biosynthesis, CYP79B2 and CYP79B3, two *Arabidopsis* cytochrome P450 enzymes, convert tryptophan to indole-3-acetaldoxime (IAOx), which is the common precursor of auxin, camalexin and indolic glucosinolates^8^,^9^,^10^, while another cytochrome P450, CYP83B1, controls the flux of IAOx to the indolic glucosinolate pathway^11^. In recent years, a group of MYB transcription factors belonging to subgroup 12 R2R3-MYB transcription factors were identified to regulate glucosinolate biosynthesis, among which MYB34, MYB51, and MYB122 distinctly regulate indolic glucosinolate biosynthesis^12^,^13^,^14^,^15^,^16^,^17^,^18^. Furthermore, diverse environmental stimuli, including wounding, pathogens, insect herbivores as well as light and nutrition, have been shown to regulate glucosinolate metabolism through MYB transcription factors^19^,^20^,^21^,^22^,^23^,^24^,^25^,^26^,^27^.

Glucosinolate accumulation has been demonstrated to be enhanced by sulfur fertilization in some cases^24^,^28^,^29^,^30^. Approximately 6% of the total sulfur in the youngest leaves of oilseed rape is assimilated into glucosinolates under sufficient sulfur supply, and glucosinolates in vegetative tissues account for 2% to 8% of the total sulfur^28^,^31^. Inorganic sulfate is the main form of sulfur taken in by plants, and firstly activated by ATP sulfurylase (ATPS) with adenylation to adenosine 5′-phosphosulfate (APS). As a branching point of sulfate assimilation, APS can be reduced by APS reductase (APR) to sulfite, which is subsequently reduced to sulfide by sulfite reductase (SiR) and finally participates in the synthesis of cysteine and other sulfur-containing compounds. In addition, APS can also be phosphorylated by APS kinase (APK) to 3′-phosphoadenosine 5′-phosphosulfate (PAPS), which donates active sulfate to the sulfation of the desulfo-GS precursors or sulfation in other secondary metabolism by sulfotransferases (SOTs)^32^,^33^,^34^,^35^,^36^,^37^. Sulfur assimilation in plants is a complex process, and is regulated by numerous factors, such as nutrients including carbon, nitrogen and sulfur, environment conditions, and phytohormones^38^,^39^,^40^,^41^,^42^,^43^,^44^,^45^,^46^.

Glucose has fundamental and multiple effects on plant metabolism at different developmental stages^47^,^48^,^49^,^50^,^51^. Glucose signaling is one of the best elucidated signaling pathways in plant cells. *Arabidopsis* hexokinase 1 (HXK1), the conserved glucose sensor with uncoupled signaling activity and phosphorylation, mediates many glucose signaling events that control the daily life of plants^48^,^52^,^53^,^54^. Recently, several reports have illustrated that sugars modulate biosynthesis of plant secondary metabolites in *Arabidopsis* and *Brassica* crops^55^,^56^,^57^,^58^. Our former study has demonstrated that glucose positively regulated aliphatic glucosinolate biosynthesis by HXK1-mediated signaling via transcription factors MYB28, MYB29, and ABA-insensitive 5 (ABI5)^59^. As another major kind of glucosinolates in *Arabidopsis*, indolic glucosinolate is synthesized via a distinct pathway from that of aliphatic glucosinolate. Here, we investigated the regulatory mechanism of indolic glucosinolate by glucose signaling, and found that glucose promotes the accumulation of indolic glucosinolates through MYB34, MYB51, and MYB122, while MYB34 plays a key role. This process is mediated by HXK1, but not by ABI5, suggesting that the mechanism underlying glucose-regulated indolic glucosinolates is distinct from that of aliphatic ones. To date, little is known about the role of glucose in sulfur assimilation or sulfur partitioning between primary and secondary sulfur metabolism. In this study, we found that glucose-promoted accumulation of both aliphatic and indolic glucosinolates is associated with enhanced sulfur assimilation.

## Results

### Glucose boosts indolic glucosinolate biosynthesis in *Arabidopsis*

The contents of three individual (I3M, 3-indolylmethyl glucosinolate; 4MOI3M, 4-methoxy-3-indolylmethyl glucosinolate; 1MOI3M, 1-methoxy-3-indolylmethyl glucosinolate) and total indolic glucosinolates were analyzed in shoots of 10-day-old Ler and Col-0 seedlings treated by 3% (W/V) of glucose or sorbitol. It was reported that different kinds of abiotic stresses including osmotic stress could affect glucosinolate content^60^,^61^,^62^, so sorbitol was used as a osmotic stress control of glucose treatment. As shown in Table 1, significant increases in the content of I3M, the predominant indolic glucosinolate composition, as well as the total indolic glucosinolates were observed in glucose-treated shoots, compared with sorbitol-treated ones. Moreover, considering that indolic glucosinolates made up a high percentage of the total glucosinolates in roots^63^, we also detected the changes of total indolic glucosinolate content in roots of seedlings treated with glucose for 3 days. Similarly, their accumulation was enhanced by glucose treatment (Fig. S1). Detailed analysis of the total content of indolic glucosinolates showed that glucose treatment dramatically increased total indolic glucosinolate accumulation in a time dependent manner, compared with sorbitol treatment (Fig. S2). Since a small gene subfamily of cytochrome P450 monooxygenases, *CYP81Fs*, were reported to convert I3M to 4MOI3M (*CYP81F2/3*) and 1MOI3M (*CYP81F4*)^64^, we examined expression levels of *CYP81F2/3/4* under glucose treatment. Consistently, transcripts of *CYP81F2/3/4* were induced by glucose, particularly of *CYP81F3* and *CYP81F4*. Taken together, our data suggest that glucose promotes the biosynthesis of indolic glucosinolates.

### Glucose induces the expression of genes related to indolic glucosinolate biosynthesis

**Figure 1:**
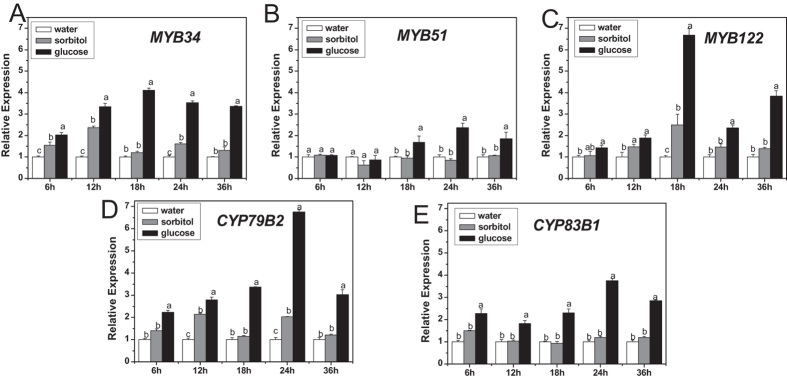
Relative expression levels of *MYB34* (**A**), *MYB51* (**B**), *MYB122* (**C**), *CYP79B2* (**D**), and *CYP83B1* (**E**) in young seedlings treated with glucose or sorbitol for indicated times. The expression level was measured in 10-day-old *Arabidopsis* seedlings treated with 3% glucose or sorbitol, and then the whole plants were collected 6, 12, 18, 24, and 36 h after treatment, respectively. Each data point represents the mean of five independent biological replicates per treatment (mean ± SE). Expression level of genes in water-treated seedlings was set to 1. The gene transcription levels upon three treatments were compared for each time point and values not sharing a common letter are significantly different at *P* < 0.05.

The response of three vital transcription factors (*MYB34*, *MYB51*, and *MYB122*) and two important biosynthetic genes (*CYP79B2*, *CYP83B1*) to glucose was analyzed. Expression of all these five genes was induced by exogenous glucose (Fig. 1). Glucose-induced mRNA levels of *MYB34*, *MYB122*, *CYP79B2*, and *CYP83B1* were detected as early as 6 h after glucose treatment and subsequently increased steadily until reaching a peak at 18 h (*MYB34*, *MYB122*) or 24 h (*CYP79B2*, *CYP83B1*). *MYB51* responded to glucose more slowly and mildly than the other two transcription factors. The mRNA levels of *MYB34*, *MYB51*, *MYB122*, *CYP79B2*, and *CYP83B1* under glucose treatment accumulated ~3.40-, 1.78-, 2.68-, 2.45-, and 2.92-folds of those in sorbitol treatment, respectively, at 18 h. Thus, plants were sampled at this time point for the following analyses of gene expression.

### Glucose-induced biosynthesis of indolic glucosinolates is affected in *myb* loss-of-function mutants

**Figure 2:**
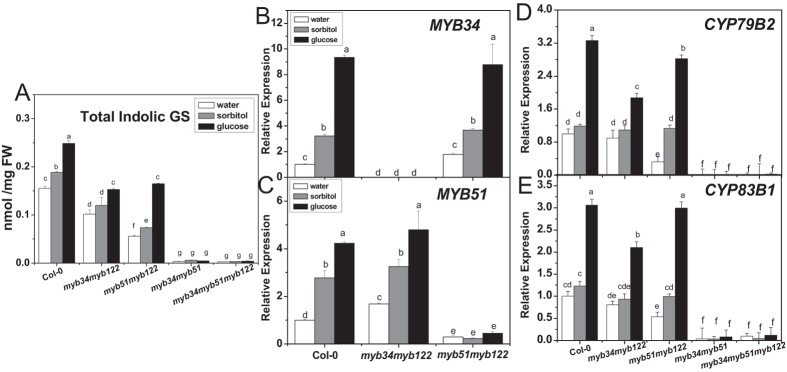
Total indolic glucosinolate content and relative expression levels of genes related to glucosinolate biosynthesis in double and triple *myb* mutant seedlings treated with glucose or sorbitol. Effect of glucose on accumulation of total indolic glucosinolates (**A**) was measured in *myb34myb122*, *myb51myb122*, *myb34myb51* double and *myb34myb51myb122* triple mutants, as well as expression levels of *MYB34* (**B**), *MYB51*(**C**), *CYP79B2* (**D**), and *CYP83B1* (**E**) in these mutants. Ten-day-old *Arabidopsis* seedlings were treated with 3% glucose or sorbitol, and then the whole plants were collected 3 days (**A**) or 18 h (**B–E**) after treatment. Each data point represents the mean of three to five (for qPCR assay) or six (for glucosinolate assay) independent biological replicates per treatment (mean ± SE). Expression level of genes in water-treated Col-0 seedlings was set to 1. Values not sharing a common letter are significantly different at *P* < 0.05.

The content of total indolic glucosinolates in *myb34myb122*, *myb51myb122*, *myb34myb51* double and *myb34myb51myb122* triple mutants was measured with or without glucose treatment. As shown in Fig. 2A, these mutants produced less indolic glucosinolates compared with the wild type under the condition without glucose. The level of indolic glucosinolates was significantly lower in *myb51myb122* than *myb34myb122*, and was almost undetected in *myb34myb51* and *myb34myb51myb122*. However, the content of total indolic glucosinolates increased by 28% in *myb34myb122* and 125% in *myb51myb122* after glucose treatment compared with sorbitol treatment, whereas glucose had no such an effect on indolic glucosinolate accumulation in *myb34myb51* and *myb34myb51myb122* mutants.

Furthermore, transcript levels of *MYB34*, *MYB51*, *CYP79B2*, and *CYP83B1* in *myb* mutants were analyzed. The expression levels of *MYB34* in *myb51myb122* and *MYB51* in *myb34myb122* were induced by glucose treatment, which was similar to that in wild type. Notably, the steady-state levels of two biosynthetic genes, *CYP79B2* and *CYP83B1* were in line with the increased levels of indolic glucosinolates (Fig. 2D,E). In addition, the expression of *CYP79B2* and *CYP83B1* in *myb51myb122* was induced almost as strong as in wild type by glucose, which was not the case for *myb34myb122*, *myb34myb51*, and the triple *myb* mutant. This observation pointed out the importance of three MYBs and especially of MYB34 in glucose-induced indolic glucosinolate biosynthesis.

### HXK1 is involved in glucose-induced indolic glucosinolate biosynthesis

**Figure 3:**
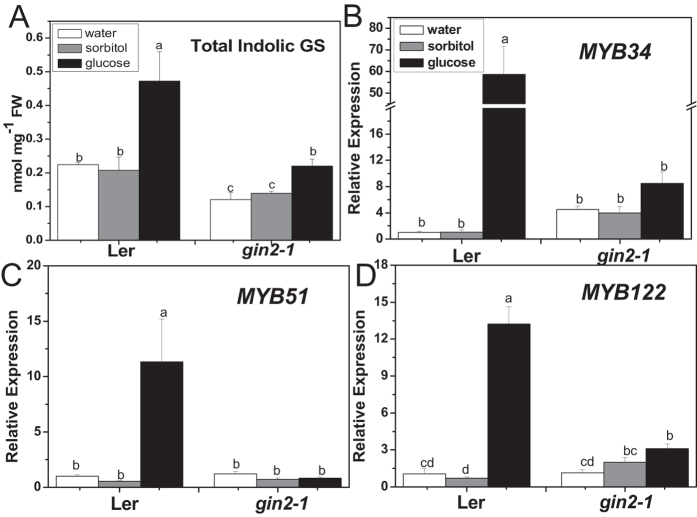
Total indolic glucosinolate content and relative expression levels of *MYB34*, *MYB51*, and *MYB122* in glucose- or sorbitol-treated *gin2-1* seedlings. Ten-day-old *Arabidopsis* seedlings were treated with 3% glucose or sorbitol, and then the whole plants were collected 3 days (**A**) or 18 h (**B–D**) after treatment. Each data point represents the mean of five (for qPCR assay) or six (for glucosinolate assay) independent biological replicates per treatment (mean ± SE). Values not sharing a common letter are significantly different at *P* < 0.05. Relative expression values are given compared with Ler seedlings treated by water.

The *gin2-1* is a HXK1 null mutant, which is insensitive to glucose^54^. As shown in Fig. 3A, *gin2-1* mutant contained a significantly reduced level of total indolic glucosinolates in comparison with the wild type. The increased accumulation of total indolic glucosinolates resulted from glucose treatment in wild type was also inhibited in this mutant. Consistently, over-expressing *HXK1* in *gin2-1* plants recovered the deficiency of glucosinolates (Fig. S4). Similarly, the induced expressions of *MYB34*, *MYB51*, and *MYB122* by glucose were almost vanished in the *gin2-1* mutant (Fig. 3B–D). All above results suggest that HXK1 is important in glucose-induced indolic glucosinolate biosynthesis.

### The role of ABI5 in glucose-induced indolic glucosinolate biosynthesis

**Figure 4:**
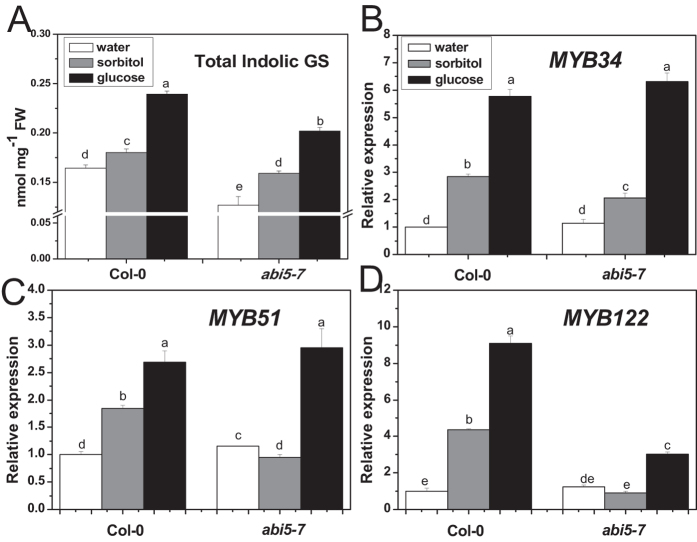
Total indolic glucosinolate content and relative expression levels of *MYB34*, *MYB51*, and *MYB122* in *abi5-7* treated with glucose or sorbitol. Ten-day-old *Arabidopsis* seedlings were treated with 3% glucose or sorbitol, and then the whole plants were collected 3 days (**A**) or 18 h (**B–D**) after treatment. Each data point represents the mean of five (for qPCR assay) or six (for glucosinolate assay) independent biological replicates per treatment (mean ± SE). Values not sharing a common letter are significantly different at *P* < 0.05. Relative expression values are given compared with Col-0 seedlings treated by water.

**Figure 5:**
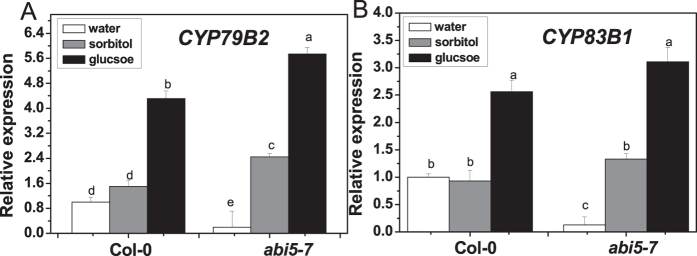
Relative expression levels of *CYP79B2* (**A**) and *CYP83B1* (**B**) in *abi5-7* treated with glucose or sorbitol. Ten-day-old *Arabidopsis* seedlings were treated with 3% glucose or sorbitol, and then the whole plantswere harvested 18 h after treatment. Each data point represents the mean of five independent biological replicates per treatment (mean ± SE). Values not sharing a common letter are significantly different at *P* < 0.05. Relative expression values are given compared with Col-0 seedlings treated by water.

The *abi5-7* mutant produced notable decreased total indolic glucosinolate content, compared with the wild type, in the presence or absence of glucose (Fig. 4A). However, glucose treatment raised the accumulation of total indolic glucosinolates in *abi5-7* by 27%, compared with sorbitol treatment, which was comparable to that in wild type (33%). To our surprise, the transcription levels of *MYB34* and *MYB51* in *abi5-7* were the same as in the wild type (Fig. 4B,C). We then checked transcription levels of *CYP79B2* and *CYP83B1* in the *abi5-7* mutant. Quantitative PCR (qPCR) analysis showed that relative expression levels of *CYP79B2* and *CYP83B1* were much lower in *abi5-7* than in the wild type (Fig. 5). However, glucose treatment dramatically enhanced the expression of these two genes in *abi5-7*, which was similar to that in the wild type, compared with sorbitol treatment.

### Glucose induces the expression of sulfate metabolic genes

**Figure 6:**
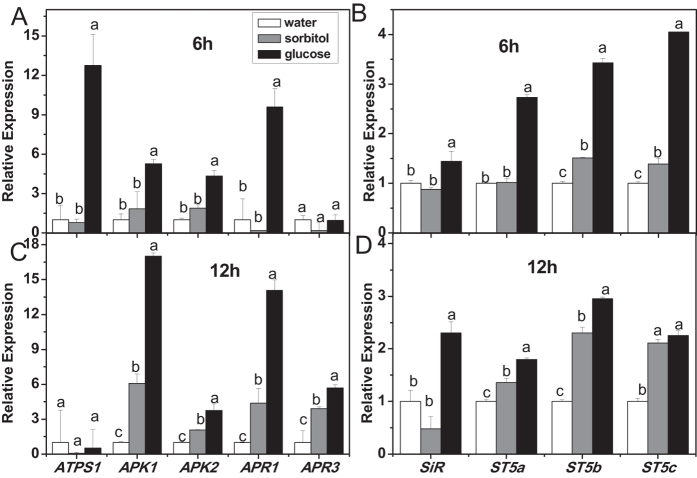
Relative expression levels of genes related to sulfate metabolism in glucose- or sorbitol-treated seedlings. Ten-day-old *Arabidopsis* seedlings were treated with 3% glucose or sorbitol, and then the whole plants were harvested 6 h (**A,B**) or 12 h (**C,D**) after treatment. Each data point represents the mean of five independent biological replicates per treatment (mean ± SE). Values not sharing a common letter are significantly different at *P* < 0.05. Relative expression values are given compared with seedlings treated by water.

Most sulfur is absorbed by plants in inorganic sulfate, and then, plants either reduce sulfate and incorporate it into cysteine and other sulfur-containing compounds of primary metabolism, or take it into the secondary metabolism to synthesize various sulfated compounds, including glucosinolates^33^,^35^. The response of sulfate metabolic genes to glucose treatment was analyzed to verify whether glucose exerted an effect on sulfate assimilation (Fig. 6). The results showed that *ATPS1* responded to glucose treatment quickly, representing a high induced expression level at 6 h, and then went back to the control level 12 h after glucose treatment. In addition, relative expression levels of *APK1*, *APK2*, *APR1*, *SiR*, and *SOTs* (*ST5a*, *ST5b*, *ST5c*) were increased by glucose as early as 6 h after treatment and maintained at a high level until 12 h. It seemed that sorbitol treatment stimulated expression of some genes, such as *ARP1* and *ST5b*, more slowly than glucose treatment (Fig. 6C,D), indicating that glucose signaling is switched on rapidly in regulating sulfate assimilation.

### Glucose increases glucosinolate accumulation and thiol content in plants cultured under different sulfate concentrations

**Figure 7:**
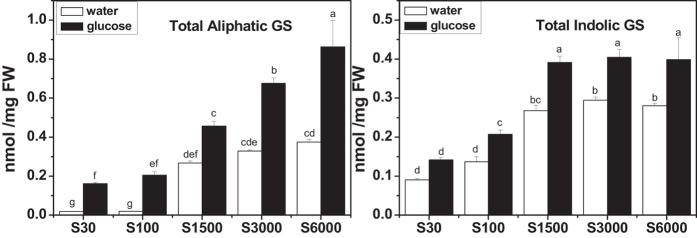
Glucosinolate contents in glucose-treated seedlings grown at different concentrations of sulfate. *Arabidopsis* seedlings (wild type Col-0) were cultured at different concentrations of sulfate for 10 days and subsequently treated with 3% glucose. The whole plants were harvested 3 days after glucose treatment. Total aliphatic and indolic glucosinolate contents were measured. Each data point represents the mean of six independent biological replicates per treatment (mean ± standard error). Values not sharing a common letter are significantly different at *P* < 0.05.

**Figure 8:**
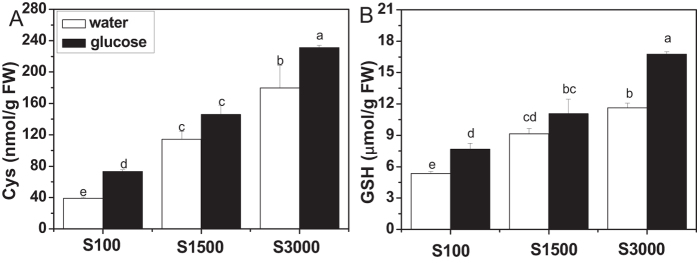
Cys and GSH contents in glucose-treated seedlings grown at different concentrations of sulfate. *Arabidopsis* seedlings (wild type Col-0) were cultured at different concentrations of sulfate for 10 days and then treated with 3% glucose. The whole plants were harvested 3 days after treatment, and then cysteine and GSH contents were measured. Each data point represents the mean of five independent biological replicates per treatment (mean ± standard error). Values not sharing a common letter are significantly different at *P* < 0.05.

To investigate whether glucose-regulated glucosinolate accumulation was associated with the concentration of sulfate, we measured the content of glucosinolates in *Arabidopsis* cultured with different concentrations of sulfate. As shown in Fig. 7, plants accumulated more glucosinolates along with the increased concentration of sulfate in culture medium in the absence of glucose. When the sulfate concentration was increased to 1500 μM, the accumulation of both aliphatic and indolic glucosinolates reached saturation. However, their levels were differentially affected by glucose. More aliphatic glucosinolates accumulated with the increased sulfate concentration. Conversely, plants synthesized almost the same amount of indolic glucosinolates when sulfate concentration reached 1500 μM or even higher. This observation suggests differential roles of both glucose and sulfur in the production of aliphatic and indolic glucosinolates. In addition, the effect of glucose on cysteine and GSH levels were assessed. As shown in Fig. 8, plants accumulated more thiols when they were supplied with more sulfate, and glucose treatment promoted thiol accumulation at all tested sulfate concentrations, especially when the concentration was slightly low (100 μM) or high (3000 μM).

## Discussion

### Glucose promotes indolic glucosinolate biosynthesis via indolic MYB transcription factors

Photosynthetic plants rely on sugars throughout their entire life, and glucose signaling is one of the vital mechanisms that control almost all phases of the plant life cycle^48^,^50^. Up to now, the mechanism of sugar-regulated anthocyanin biosynthesis has been well illustrated, while knowledge about sugar-regulated glucosinolate biosynthesis is limited^55^,^56^,^57^,^58^,^65^,^66^,^67^. Our former work indicated a positive role of glucose in promoting aliphatic glucosinolate biosynthesis^59^. In the present study, we found that glucose enhanced indolic glucosinolate accumulation in different parts (shoots and roots) of *Arabidopsis* seedlings with different genetic backgrounds (Col-0 and Ler) (Table 1, Fig. S1). Besides, glucose treatment could induce the expression of *CYP81Fs* (Fig. S3), indicating that glucose promoted the conversion of I3M to both 1MOI3M and 4MOI3M. Thus, our data reveals that glucose has a broad influence on glucosinolate biosynthesis.

MYB28 and MYB29 are two major transcription factors controlling aliphatic glucosinolate biosynthesis in *Arabidopsis*, of which MYB28 plays a key role^14^,^17^,^18^,^68^. Similarly, MYB28 acts predominantly in glucose-induced aliphatic glucosinolate accumulation^59^. Frerigmann and Gigolashvili (2014) recently addressed the distinct functions of MYB34, MYB51, and MYB122 in indolic glucosinolate biosynthesis^13^. In the present study, the expression of *MYB34, MYB51*, and *MYB122* was induced by glucose, with that of *MYB34* being the highest, compared with sorbitol treatment (Fig. 1). Both *myb34myb51* double and *myb34myb51myb122* triple mutants (with the absence of MYB34 and MYB51) produced only traces of indolic glucosinolates even in the presence of glucose (Fig. 2), indicating that MYB34 and MYB51 were crucial for the basal indolic glucosinolate biosynthesis. Interestingly, although *myb51myb122* contained less indolic glucosinolates than *myb34myb122*, a much stronger induction of indolic glucosinolate biosynthesis occurred in *myb51myb122* upon glucose treatment. This finding substantiated a special role of MYB34 in glucosinolate biosynthesis in response to glucose. Remarkably, the specific response of various MYB transcription factors to environmental stimuli is a universal phenomenon. Among aliphatic MYBs, MYB28 is vital in glucose signaling, whereas MYB29 plays an important role in response to jasmonic acid (JA) and salicylic acid (SA)^17^,^18^. As for indolic glucosinolate, MYB51 is known to be crucial in ethylene and SA-mediated induction of their biosynthesis in addition to the transient response to the mechanical stimulus^13^,^15^. MYB34 is known as the key regulator in response to abscisic acid (ABA)^13^, as well as glucose signaling as shown by our present study. Taken together, we have demonstrated that glucose enhanced indolic glucosinolate biosynthesis through indolic MYBs, in which MYB34 and MYB51 were crucial in maintaining the basal indolic glucosinolate accumulation, while MYB34 was pivotal in response to glucose signaling.

### Regulation of indolic glucosinolate biosynthesis by glucose is HXK1-dependant

*Arabidopsis* HXK1, the intracellular glucose sensor, functions in various glucose-regulated processes. The role of HXK1 in sensing glucose signal was uncoupled from glucose metabolism^54^,^69^,^70^,^71^,^72^. It has been known that the repression of glucose on ethylene response factor 1 (*ERF1*) and activation on biosynthetic gene *ABA2*, transcription factor *ABI4* are HXK1 dependent^52^,^70^,^73^. In the present study, expression of *ABI5* greatly induced by glucose in the wild type disappeared in *gin2-1* (Fig. S5), which was similar to the observation by Cho *et al*.^70^, suggesting that induction of *ABI5* by glucose is HXK1-dependent. Yanagisawa *et al*. (2003) reported that the degradation of ethylene-insensitive3 (EIN3), a key transcriptional regulator in ethylene signaling, was enhanced by glucose through HXK1^72^. Meanwhile, there also exists HXK1-independent glucose signaling pathways^73^,^74^,^75^. Our previous study has proved that the regulation of aliphatic glucosinolate biosynthesis by glucose was HXK1-dependent. In current survey, a notable deficiency of indolic glucosinolates was observed in *gin2-1* in comparison with the wild type, and the significant increase in indolic glucosinolate accumulation upon glucose treatment in the wild type was also inhibited in *gin2-1* (Fig. 3A), which could be rescued by overexpression of *AtHXK1* (Fig. S4). These results suggest the involvement of HXK1 in glucose-enhanced indolic glucosinolate biosynthesis. Furthermore, the increased expression of *MYB34*, *MYB51*, and *MYB122* in response to glucose was disturbed by the absence of HXK1 (Fig. 3B–D), suggesting that HXK1 also modulates the glucose-activated indolic MYBs expression. Taken together, HXK1 plays an important role in glucose-induced indolic glucosinolate biosynthesis.

### ABI5 participates in indolic glucosinolate biosynthesis, but is not required for glucose-induced indolic glucosinolate biosynthesis

*ABI5* encodes a transcription factor belonging to a large basic leucine zipper (bZIP) domain family, and plays a crucial role in ABA signal transduction, especially during seed development and germination^76^,^77^,^78^,^79^. The *abi5-7* mutant is originally discovered for its insensitivity to high level of exogenous ABA (3 μM), which is normally inhibitory for seed germination and seedling development of the wild type^80^. However, it is also found to be insensitive to glucose, revealing a function of ABI5 in glucose signaling^76^,^81^,^82^. Furthermore, the expression of *ABI5* is greatly increased by low concentrations of glucose in the wild type, but not in *gin2-1*, indicating that the regulation of *ABI5* by glucose is HXK1-dependent^70^ (Fig. S5). In our former survey, ABI5 is proved to be involved in the regulation of aliphatic glucosinolate biosynthesis as a mediator in glucose signaling^59^. However, it seems that ABI5 acts in a different way in regulation of indolic glucosinolate biosynthesis. Undoubtedly, ABI5 participates in indolic glucosinolate biosynthesis based on the decreased constitutive indolic glucosinolate content and decreased expression levels of biosynthetic genes (*CYP79B2*, *CYP83B1*) in the *abi5-7* mutant (Figs 4A and 5). Nevertheless, the absence of ABI5 did not interfere with the glucose-triggered increase in indolic glucosinolate biosynthesis and expression of biosynthetic genes (Figs 4A and 5), suggesting that ABI5 was not required for glucose-induced indolic glucosinolate biosynthesis. In addition, both the basal mRNA level of indolic MYB transcription factors and the inducing effect of glucose on their expression were not disturbed in *abi5-7* (Fig. 4B–D). This observation points out the possibility that regulation occurs at a protein level or with other factors. Recently, MYC2, MYC3, and MYC4 were shown to form complexes with MYBs to modulate glucosinolate biosynthesis^83^,^84^. The possibility of existing ABI5-MYB complexes in regulating indolic glucosinolate biosynthesis or post-transcriptional control of indolic MYBs by ABI5 remains to be verified. Further studies are needed to elucidate the regulatory mechanism and to identify the yet unknown factors involved in the regulation of indolic glucosinolate biosynthesis by glucose signaling.

### Glucose enhances indolic glucosinolate accumulation without weakening primary sulfate assimilation

Sulfur is an essential nutrient for all higher plants, and plants reduce sulfate to sulfide and incorporate it into organic metabolites^85^. The backbone of glucosinolates contains from two to three S atoms, with one originating from 3′-phosphoadenosine 5′-phosphosulfate, the second one from glutathione, and the third being present in methionine derived aliphatic glucosinolates^86^. Sulfur assimilation is therefore crucial for glucosinolate biosynthesis^35^,^36^. Up to now, great progress has been achieved in illustrating the regulation of sulfur metabolism^32^,^33^,^87^,^88^. The effect of glucose on sulfur assimilation has also been investigated^38^,^41^,^42^. Hesse *et al*. (2003) reported that 0.5% (w/v) glucose induced an increase in the APR level and incorporation of S in *Arabidopsis*^38^. There are also reports demonstrating that the addition of sucrose or glucose led to a rise in the mRNA level and enzyme activity of APR^41^,^42^. In the present study, our results showed that glucose treatment increased the mRNA levels of sulfate metabolism related genes, such as *ATPS1*, *APK1*, *APK2*, *APR1*, *SiR*, *ST5a*, *ST5b*, and *ST5c* (Fig. 6), indicating a positive effect of glucose on sulfur assimilation. As shown in Fig. 7, an increase in glucosinolate content was observed in plants grown in higher sulfate concentrations, which was accordant with descriptions of other reports^24^,^28^,^29^,^30^. The glucosinolate accumulation reached saturation when sufficient sulfur (about 1500 μM) was given to the plants. Interestingly, addition of glucose to the media with sufficient sulfur could further enhance glucosinolate biosynthesis, which could be explained by the simultaneous activation of sulfur assimilation and glucosinolate biosynthesis^59^ (Figs 1 and 6). However, the accumulation of aliphatic and indolic glucosinolate in response to glucose induction was under different mechanism. As shown in Fig. 7, the biosynthesis of aliphatic glucosinolates could be further induced by adding both glucose and sulfate in the media while indolic glucosinolate biosynthesis could not be further stimulated by the addition of glucose or sulfate when its content reached about 0.4 nmol/mg fresh weight (FW). Aliphatic glucosinolates can be synthesized from methionine, the production of sulfate assimilation. Hence there are sufficient precursors for aliphatic glucosinolate biosynthesis as sulfate assimilation in plants can be enhanced by glucose treatment. In contrast, the precursor of indolic glucosinolate is tryptophan. It has been report that, in case of S starvation, the pools of reduced sulfur containing organic molecules (such as cysteine and GSH) decreased, while the precursors (O-acetylserine and serine) accumulated^32^,^89^,^90^. Moreover, as serine is directly involved in tryptophan biosynthesis^91^, and tryptophan showed evident elevated levels under sulfur-deficient growth conditions^89^,^90^,^92^. Plant usually delicately balances the nutrients or metabolites. When S is sufficient, serine might be utilized for biosynthesis of immediate products, resulting in a competition with the conversion of serine to tryptophan. Therefore, indolic glucosinolates could not be constantly accumulated. The analysis of thiol content showed that glucose treatment also enhanced the synthesis of cysteine and GSH, the productions of primary sulfate assimilation (Fig. 8A,B). Hence, the induction of glucosinolate accumulation by glucose should be mainly derived from the enhanced sulfate assimilation, but not the sulfur partitioning into glucosinolate biosynthesis. As glucose could activate sulfur assimilation and enhance thiol synthesis, we further investigated the role of HXK1, the receptor of glucose signaling in this process. Interestingly, glucose-modulated expressions of *APK1*, *SiR*, and *ST5c* were strongly inhibited in *gin2-1* mutant (Fig. S6B,D,E). However, a strong induction of *ATPS1* expression by glucose was also observed in this mutant, and no significant difference existed in expression of *APR1* between *gin2-1* mutant and the related wild type plants (Fig. S6A,C). These results demonstrated that glucose-regulated sulfur assimilation is only partially HXK1-dependent. The regulation of glucose in primary sulfur assimilation is complicated, and further studies are needed to elucidate the mechanism.

It has been reported that glucose activates ABA biosynthesis and signal transduction via transcription factor^52^,^70^,^73^. The function of ABA in promoting indolic glucosinolate accumulation^13^ and enhancing cysteine and GSH synthesis has also been demonstrated^39^,^93^. Therefore, whether the effect of glucose on glucosinolate and thiol synthesis is ABA-dependent needs to be verified in the future.

**Figure 9:**
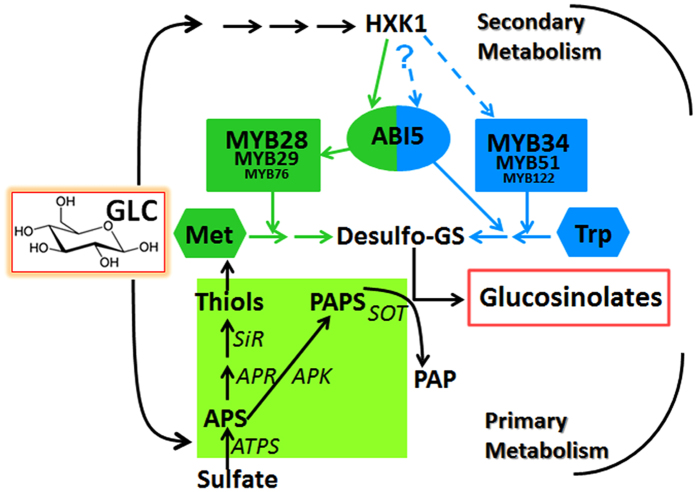
A model for glucose-regulated glucosinolate biosynthesis in *Arabidopsis*. Glucose plays a key and positive role in both primary and secondary metabolism. Symbols with light green color represent sulfate metabolism pathway. Symbols with dark green color represent aliphatic glucosinolate biosynthesis and regulation. Symbols with blue color represent indolic glucosinolate biosynthesis and regulation. Multiple enzymatic steps are indicated with interrupted arrows. GLC, glucose; Met, methionine; Trp, tryptophan; Desulfo-GS, Desulfo-glucosinolates; ^– – – –**>**^ possible activation.

We summarize our present and former work^59^ as Fig. 9, which suggests a positive role of glucose in both glucosinolate biosynthesis and sulfur assimilation. Glucose promotes glucosinolate biosynthesis mainly through MYB28 and MYB34 in a HXK1 dependent manner. However, the regulation by ABI5 in glucose signaling is distinct between aliphatic and indolic glucosinolate biosynthesis. ABI5 seems to be involved in biosynthesis of both aliphatic and indolic glucosinolates, as well as in glucose-induced aliphatic rather than indolic glucosinolate biosynthetic pathway. The diverse biological function of aliphatic and indolic glucosinolates in plant development and stress resistance might explain the significance of their biosynthesis under different regulatory mechanisms by the same glucose signaling. In addition, glucose enhances glucosinolate biosynthesis by strengthening sulfur assimilation instead of simply redirecting sulfur partitioning into glucosinolate biosynthesis.

## Materials and Methods

### Plants and Growth Conditions

The sterilized seeds were stratified for 3 days at 4 °C, and transferred into flasks (~50 seeds per flask) with 40 ml of liquid growth medium [full-strength sterilized Murashige–Skoog (MS) salt solution +0.5% glucose]. For assays in *Arabidopsis* cultured at different concentrations of sulfate, the liquid growth medium was modified Murashige–Skoog (MS) salt solution +0.5% glucose. S0 was prepared by complete replacement of SO_4_^2−^ to Cl^−^. S30, S100, S1500, S3000, and S6000 liquid media were prepared by adding MgSO_4_ to the S0 medium at final concentrations of 30 μM, 100 μM, 1500 μM, 3000 μM, and 6000 μM. Mg^2+^ concentration was adjusted to 1500 μM by adding MgCl_2_^94^. Plants were grown under a photoperiod of 16 h light/8 h dark with gentle shaking (135 rpm) for 10 days in a plant growth chamber at 21 °C^55^. The *gin2-1* and *abi5-7* mutants were generously provided by Dr. Sheng Teng (Shanghai Institute of Plant Physiology and Ecology, Chinese Academy of Sciences). The double mutant *myb34myb51*, *myb34myb122*, *myb51myb122*, and triple mutant *myb34myb51myb122* were isolated in previous study at University of Cologne, Germany^13^. The transgenic plants *AtHXK1/gin2*, over expression of *AtHXK1* in *gin2-1* background, is kindly gifted by Dr. Jen Sheen (Department of Molecular Biology and Centre for Computational and Integrative Biology, Massachusetts General Hospital, and Department of Genetics, Harvard Medical School). The genetic background of all mutants was Columbia (Col-0), except for *gin2-1*, which was Landsberg (Ler).

### Glucose and Sorbitol Treatments

Sterilized glucose and sorbitol were added into the flasks after 10 days with water as a control. 3% (w/v) was selected as the final concentration for glucose and sorbitol treatments according to our previous study^59^. After treatments, plants were cultured in the same condition as before and were collected at different time points for analysis.

### Glucosinolate Assay

The analysis of glucosinolate content was performed on a HPLC device (Shimadzu, Kyoto, Japan) as described recently^59^.

### RNA Isolation and Expression Analysis

The isolation of RNA and expression analysis were performed as previously described^59^. The expression level of *Arabidopsis ACTIN2* was used as an internal control and the expression of other genes was computed with the 2^−ΔΔCT^ method^95^. Primers are listed in Table S1.

### HPLC Analysis of Thiols

The analysis of cysteine and glutathione (GSH) was performed as described^37^. Approximately 100 mg of plant materials were homogenized in liquid nitrogen. After adding 1 ml of cold 0.1 M HCl, samples were mixed and agitated at room temperature at 300 rpm for 40 min. The suspension was centrifuged at 14000 rpm for 20 min at 4 °C. 120 μl of supernatant were mixed with 200 μl of 0.25 M CHES-NaOH, pH 9.4 and 70 μl of 10 mM dithiothreitol by vortexing, and then incubated at room temperature for 40 min. After that, 10 μl of 25 mM of monobromobimane were added and the derivatisation of thiols was conducted by incubating samples in the dark at room temperature for 15 min. Reaction was stopped by adding 220 μl of 100 mM methanesulfonic acid. After 30 min centrifugation at 14000 rpm, 4 °C, supernatants were collected for HPLC analysis.

The separation and analysis of the thiols were performed using reversed-phase (Hypersil C18 column, 5 μm particle size, 4.6 mm × 250 mm; Elite Analytical Instruments Co. Ltd, Dalian, China) HPLC system. 10% (v/v) methanol mixed with 0.25% (v/v) acetic acid (pH 3.9) was set as solvent A and 90% (v/v) methanol mixed with 0.25% (v/v) acetic acid (pH 3.9) as solvent B. The flow rate was kept constant at 1.0 ml min^−1^. The procedure employed isocratic elution with 100% solvent A for the first 2 min; a linear gradient to 92% over the next 10 min; followed by a linear gradient to 86% within 5 min. Bimanederivates were detected fluorimetrically (Shimadzu, Kyoto, Japan) with excitation at 390 nm and emission at 480 nm.

### Statistical Analysis

Statistical analysis was performed using the SPSS package program version 11.5 (SPSS Inc., Chicago, IL, USA). Differences were analyzed by one-way analysis of variance (ANOVA), followed by the least significant difference (LSD) test at a 95% confidence level (*P* < 0.05). The values are reported as means with standard error for all results.

## Additional Information

**How to cite this article**: Miao, H. *et al*. Glucose enhances indolic glucosinolate biosynthesis without reducing primary sulfur assimilation. *Sci. Rep*. **6**, 31854; doi: 10.1038/srep31854 (2016).

## Supplementary Material

Supplementary Information

## Figures and Tables

**Table 1:** Individual and total indolic glucosinolate contents (nmol/mg FW) in Ler and Col-0 treated with glucose or sorbitol.

	Ler			Col-0		
	water	sorbitol	glucose	water	sorbitol	glucose
I3M	0.072 ± 0.014b	0.069 ± 0.006b	0.191 ± 0.023a	0.070 ± 0.005b	0.079 ± 0.005b	0.165 ± 0.027a
4MOI3M	0.028 ± 0.014b	0.035 ± 0.008b	0.059 ± 0.004a	0.007 ± 0.001c	0.010 ± 0.002b	0.012 ± 0.002a
1MOI3M	0.030 ± 0.006a	0.040 ± 0.005a	0.032 ± 0.007a	0.025 ± 0.003a	0.021 ± 0.006a	0.027 ± 0.004a
Total indolic GS	0.130 ± 0.057b	0.144 ± 0.034b	0.282 ± 0.037a	0.102 ± 0.003b	0.110 ± 0.007b	0.204 ± 0.028a

Ten-day-old *Arabidopsis* seedlings were treated with 3% glucose or sorbitol. Shoots were collected after treatment for 3 days. Each data represents the mean of six independent biological replicates per treatment (mean ± SE). Values not sharing a common letter are significantly different at *P* < 0.05.
