# Supplementary material for: Glucose enhances indolic glucosinolate biosynthesis without reducing primary sulfur assimilation
Source: Sci Rep. 2016 Aug 23;6:31854. doi: 10.1038/srep31854 (PMC4994012; doi:10.1038/srep31854)
Supplement: Supplementary Information [file srep31854-s1.doc]

**Glucose enhances indolic glucosinolate biosynthesis without reducing primary sulfur assimilation** Huiying Miao^1,2^, Congxi Cai^1,2^, Jia Wei^1^, Jirong Huang^3^, Jiaqi Chang^1,2^, Hongmei Qian^1,2^, Xin Zhang^1,2^, Yanting Zhao^1^, Bo Sun^1^, Bingliang Wang^1,2^, Qiaomei Wang^1,2^**^*^**

**Supplementary Information**

**Table S1.** Primer sequences used for qPCR.

| **Target gene** | | **Locus code** | | **Primer sequence** | |
| --- | --- | --- | --- | --- | --- |
| ***ACTIN2*** | **A****T3G18780** | | **5’-TAACTCTCCCGCTATGTATGTCGC-3’**  **5’-CCACTGAGCACAATGTTACCGTAC-3’** | |  |
| ***MYB34*** | [**AT5G60890**](http://www.arabidopsis.org/servlets/TairObject?type=locus&id=134566) | | **5’-CGGGTCTTAAGTAATTAGCC-3’**  **5’-AAGAAAGGAGCTTGGACTCC-3’** | |  |
| ***MYB51*** | **AT1G18570** | | **5’-ACAAATGGTCTGCTATAGCT -3’**  **5’-CTTGTGTGTAACTGGATCAA -3’** | |  |
| ***MYB122*** | **AT1G74080** | | **5’-TCCGTTGAGTCTTGTTTGGA-3’**  **5’-TTGTCAATCCCTTCACAGGA-3’** | |  |
| ***CYP79B2*** | **AT4g39950** | | **5’-GTAACTTCGGAGCATTCGT-3’**  **5’-TCGCCGGATATCACATCC -3’** | |  |
| ***CYP83B1*** | **AT4g31500** | | **5’- TCACGCCATATCTACCAGC -3’**  **5’-TGGACGTCATGACTGGAC -3’** | |  |
| ***ATPS1*** | **AT3g22890** | | **5’- CCCGCCAGACGGTTTTATGT -3’**  **5’- ACTTCTGGTAGTCTACCATTACCGC -3’** | |  |
| ***APK1***  ***APK2***  ***APR1***  ***APR3***  ***SiR***  ***ST5a***    ***ST5b*** | **AT2G14750**  **AT4G39940**  **AT4G04610**  **AT4G21990**  **AT5G04590**  [**AT1G74100**](http://arabidopsis.org/servlets/TairObject?id=30118&type=locus)  [**AT1G74090**](http://arabidopsis.org/servlets/TairObject?id=30121&type=locus) | | **5’- CCTTACGAGCCACCATTGAACTG -3’**  **5’- GCCATTTCGATAGGAGAAGTTCCT -3’**  **5’- CAAAATCAAAGGCTTCACTGGAATC -3’**  **5’- TGTTTCAGCACTACCTCGCAATT -3’**  **5’- CGATCAAGTATCCGTCTGAGAAG -3’**  **5’- GACTTCGTTCTTGAATCTTGTCC -3’**  **5’- CCAATCAAGTATCCATCAGAGAAG -3’**  **5’- CATCTTTCTTGAATCTTGTTCGG -3’**  **5’- AATATGGGTAGCACGCTTGG -3’**  **5’- CGAGCCTTCACTACCTCTGG -3**  **5’- TTACGCAATCGTCAATCGTT -3’**  **5’- GCGAAGTCGATCTCAACGTA** **-3**  **5’-TCCATGTGGACTTTCCTTCA-3**  **5’- TCCAATACGCCAGGATATGA -3** | |  |
| ***ST5c*** | [**AT1G18590**](http://arabidopsis.org/servlets/TairObject?id=29504&type=locus) | | **5’- TCACTGGTGGCTACAACCTC -3**  **5’- TTGCGATTGCGAAAGTTAAG -3** | |  |

**Figure S1 Total indolic glucosinolate content in the roots of glucose- or sorbitol-treated seedlings.**

Ten-day-old *Arabidopsis* seedlings (wild type Col-0) were treated with 3% glucose or sorbitol. Roots were collected 3 days after treatment. Each data point represents the mean of six independent biological replicates per treatment (mean ± standard error). Values not sharing a common letter are significantly different at *p* < 0.05.

**Figure S2 Total indolic glucosinolate content in seedlings treated with glucose or sorbitol for indicated times.**

Total indolic glucosinolates content was measured in 10-day-old *Arabidopsis* seedlings (wild type Col-0) treated with 3% glucose or sorbitol, and then the whole plants were collected 0 h, 6 h, 12 h, 24 h and 36 h after treatment. Each data point represents the mean of six independent biological replicates per treatment (mean ± SE). Values not sharing a common letter are significantly different at *p* < 0.05.

**Figure S3 Relative expression levels of *CYP81Fs* in seedlings treated with glucose or sorbitol.**

Ten-day-old *Arabidopsis* seedlings were treated with 3% glucose or sorbitol, and then the whole plants were collected 18 h after treatment. Each data point represents the mean of three independent biological replicates per treatment (mean ± SE). Expression level of genes in water-treated seedlings was set to 1. Values not sharing a common letter are significantly different at *p* < 0.05.

**Figure S4 Glucosinolate contents in Ler and *AtHXK1/gin2* treated with glucose or sorbitol.**

Ten-day-old *Arabidopsis* seedlings were treated with 3% glucose or sorbitol and then the whole plants were collected 3 days after treatment. Each data point represents the mean of six independent biological replicates per treatment (mean ± standard error). Values not sharing a common letter are significantly different at *p* < 0.05.

**Figure S5 Relative expression level of *ABI5* in *gin2-1* treated with glucose or sorbitol*.***

Ten-day-old *Arabidopsis* seedlings were treated with 3% glucose or sorbitol, and then the whole plants were collected 18 h after treatment. Each data point represents the mean of three independent biological replicates per treatment (mean ± SE). Expression level of genes in water-treated seedlings was set to 1. Values not sharing a common letter are significantly different at *p* < 0.05.

**Figure S6 Effect of glucose on expression of genes related to sulfate metabolism in *gin2-1* mutant.**

The transcription levels of *ATPS1* (A), *APK1* (B), *APR1* (C), *SiR* (D), and *ST5c* (E) in response to glucose in *gin2-1* mutant. 10-day-old *Arabidopsis* seedlings were treated with 3% glucose or sorbitol, and then the whole plants were harvested at 6 h after treatment. Each data point represents the mean of three independent biological replicates per treatment (mean ± SE). Values not sharing a common letter are significantly different at *P* < 0.05. Relative expression values are given compared with Ler seedlings treated by water.
